# Modelling the geographical spread of HIV among MSM in Guangdong, China: a metapopulation model considering the impact of pre-exposure prophylaxis

**DOI:** 10.1098/rsta.2021.0126

**Published:** 2022-01-10

**Authors:** Fengshi Jing, Yang Ye, Yi Zhou, Hanchu Zhou, Zhongzhi Xu, Ying Lu, Xiaoyu Tao, Shujuan Yang, Weibin Cheng, Junzhang Tian, Weiming Tang, Dan Wu

**Affiliations:** ^1^ Institute for Healthcare Artificial Intelligence, Guangdong Second Provincial General Hospital, Guangzhou 510317, People’s Republic of China; ^2^ University of North Carolina Project-China, Guangzhou 510085, People’s Republic of China; ^3^ School of Data Science, City University of Hong Kong, Hong Kong SAR, People’s Republic of China; ^4^ Faculty of Medicine, Macau University of Science and Technology, Macau SAR, People’s Republic of China; ^5^ Zhuhai Center for Diseases Control and Prevention, Zhuhai 519060, People’s Republic of China; ^6^ School of Traffic and Transportation Engineering, Central South University, Changsha 410075, People’s Republic of China; ^7^ The Hong Kong Jockey Club Centre for Suicide Research and Prevention, The University of Hong Kong, Hong Kong SAR, People’s Republic of China; ^8^ West China School of Public Health, Sichuan University, Chengdu 610041, People’s Republic of China; ^9^ Department of Clinical Research, London School of Hygiene and Tropical Medicine, London WC1E 7HT, UK

**Keywords:** HIV transmission, human mobility data, social media data, sexual behaviour data, MSM

## Abstract

Men who have sex with men (MSM) make up the majority of new human immunodeficiency virus (HIV) diagnoses among young people in China. Understanding HIV transmission dynamics among the MSM population is, therefore, crucial for the control and prevention of HIV infections, especially for some newly reported genotypes of HIV. This study presents a metapopulation model considering the impact of pre-exposure prophylaxis (PrEP) to investigate the geographical spread of a hypothetically new genotype of HIV among MSM in Guangdong, China. We use multiple data sources to construct this model to characterize the behavioural dynamics underlying the spread of HIV within and between 21 prefecture-level cities (i.e. Guangzhou, Shenzhen, Foshan, etc.) in Guangdong province: the online social network via a gay social networking app, the offline human mobility network via the Baidu mobility website, and self-reported sexual behaviours among MSM. Results show that PrEP initiation exponentially delays the occurrence of the virus for the rest of the cities transmitted from the initial outbreak city; hubs on the movement network, such as Guangzhou, Shenzhen, and Foshan are at a higher risk of ‘earliest’ exposure to the new HIV genotype; most cities acquire the virus directly from the initial outbreak city while others acquire the virus from cities that are not initial outbreak locations and have relatively high betweenness centralities, such as Guangzhou, Shenzhen and Shantou. This study provides insights in predicting the geographical spread of a new genotype of HIV among an MSM population from different regions and assessing the importance of prefecture-level cities in the control and prevention of HIV in Guangdong province.

This article is part of the theme issue ‘Data science approach to infectious disease surveillance’.

## 1. Introduction

Despite substantial progress in tackling the human immunodeficiency virus (HIV) epidemic, HIV continues to pose public health threats in China [1,2]. Specifically, HIV transmission among men who have sex with men (MSM) has increased markedly in recent years, making up the majority of new diagnoses among young people in China [3–5]. HIV mutates frequently. Some genotypes may become more infectious and virulent than the existing ones, which causes current antiretroviral treatment (ART) to be less effective and a larger number of people to be infected [6,7]. Increasing connectivity among cities has further complicated HIV transmission. Understanding transmission patterns of a new genotype of HIV among MSM, especially the geographical spread patterns, is important for the control and prevention of HIV infections. An example of the outbreak of a new genotype in recent years is HIV-1 CRF55_01B, which was first reported in 2012 and was later proven to have originated in MSM in Shenzhen, a city of Guangdong province in China [6,7]. This genotype has been causing thousands of HIV infection cases in the whole Guangdong province through an inter-city geographical transmission pattern from Shenzhen. A surveillance system to alert neighbouring cities about the emergence of a new HIV genotype may help us better control and prevent HIV transmission across cities. Therefore, the motivation of this study is to first construct such an alarming system (i.e. an inter-city probabilistic transmission network model) for cities by hypothesizing the emergence of a new HIV genotype in a certain city and to predict possible geographical spread using a metapopulation model.

Metapopulation models are widely used to investigate the transmission of infectious diseases, such as influenza, the 2003 SARS epidemic, and the COVID-19, in a large-scale spatial area, where disease transmission among spatially separated populations occurs via population movement [8–12]. Previous research has used metapopulation models to measure the effect of human mobility on HIV transmission, to evaluate the effectiveness of different intervention strategies, and to predict further transmission [13–17]. For example, Megan *et al.* employed the metapopulation approach to estimate the impact of migration on the spread of HIV based on a population study of migrant, non-migrant men, and their rural partners, in South Africa [13]. They concluded that migration influences HIV spread by increasing likelihood of high-risk sexual behaviours in their destination cities. Xiao *et al.* assessed the HIV epidemic in mainland China based on a mobility network of diagnosed HIV/AIDS cases, which was constructed using differences in their current residences and registered residences in China surveillance systems [14]. Isdory *et al.* built a Susceptible-Infected-Removed (SIR) based metapopulation model to analyse the impact of human mobility on HIV transmission based on the mobile phone location data in Kenya [15]. They showed that mobility between regions played a relatively small role in HIV transmission in Kenya where HIV has already been endemic.

However, little has been done to examine the geographical spread of HIV among the MSM population under the context where HIV prevention measures are provided to at-risk populations, such as HIV pre-exposure prophylaxis (PrEP) in China [18]. Effective HIV prevention measures including sexuality education, condom use promotion, and PrEP can significantly lower risks for transmitting the HIV virus. We focus on the MSM population and the usage of PrEP in this model due to the high HIV prevalence among MSM in China and the fairly large number of Chinese MSM who are willing to take PrEP [19]. To investigate the geographical spread of a new HIV genotype among MSM, we construct a movement probabilistic network to infer the MSM movement patterns between cities in Guangdong, one of the major provinces with a high HIV incidence among MSM in China [20]. Based on this network, we propose a metapopulation model, considering the impact of PrEP and parameterized with sexual behaviour data among MSM in Guangdong, to mimic the HIV transmission patterns between and within cities. This study aims to predict the geographical spread of a new genotype of HIV among MSM populations from different cities, and to assess the importance of each city in the control and prevention of HIV infections through simulations considering the impact of PrEP. Note that our approach can be adopted to predict geographical spread at a larger scale (such as transmission across provinces and countries). In this paper we used inter-city geographical spread in Guangdong province to illustrate the model building process.

## 2. Inter-city movement probabilistic network

**Figure 1.**
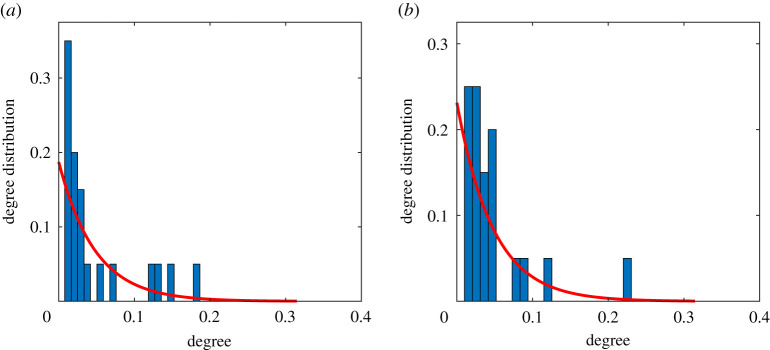
Weighted degree distribution of (*a*) the offline mobility network and (*b*) the online social network. For clear illustration, the weighted degree of each city is rescaled by dividing through the sum of the weighted degree of all cities. (Online version in colour.)

**Figure 2.**
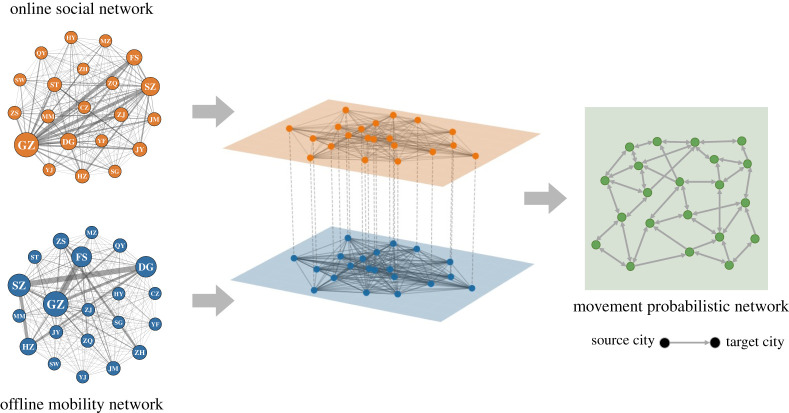
Construction of the movement probabilistic network. The panels in the left column show the visualizations of the online social network (top one; obtained from BlueD) and the offline mobility network (bottom one; obtained from Baiduqianxi). A node represents a city in Guangdong, China. Node labels represent the abbreviations of city names. A detailed mapping of city names and abbreviations is presented in the electronic supplementary material. An edge on online social network represents the number of follower–followee relationships between two cities. An edge on the offline mobility network represents the number of individuals travelling between two cities. The movement probabilistic network on the right column is constructed by integrating the information on the online social network and the offline mobility network (the middle panel); see equation (2.1). A directed edge on the movement probabilistic network represents the probability of individuals moving from the source city to the target city. (Online version in colour.)

Because (i) MSM with follower–followee relationships in online social media may have real-life contacts [21], and (ii) MSM population may have similar mobility patterns to other populations, we constructed an inter-city movement probabilistic network among MSM which integrates the multi-source data of online social networks and the offline mobility network to capture the MSM movement patterns between 21 prefecture-level cities (we use ‘cities’ thereafter for simplicity) in Guangdong. The offline mobility network is constructed using data from Baiduqianxi [22], a public website providing a real-time migration index based on the number of inbound and outbound events by rail, air and road traffic across mainland China for the entire migrant population (not just MSM migrants). Here, a node represents a city. An edge between nodes represents the average number of individuals travelling between two cities. We specify the number of MSM travelling between cities on a pro rata basis: denote the total number of migrants travelling between city i and city j per time step as $M_{ij}$, then the number of MSM migrants travelling between city i and city j per time step is $\delta M_{ij}$, where δ denotes the percentage of MSM population among the whole population for all cities (see electronic supplementary material for details). The online social network is constructed using data from Blued, the biggest gay men's dating app in China [23]. We randomly select 2011 users located in Guangdong province to infer the general online inter-city social network. Here, a node also represents a city. An edge between two cities represents the number of follower–followee relationships between two cities. Figure 1 illustrates the weighted degree distribution of the offline mobility network and the online social network. We can observe the long-tailed (power-low) degree distribution on both networks, and the offline mobility network has a longer tail than the online social network. We illustrate the construction of the movement probabilistic network in figure 2. We denote $X_{ij}$, $Y_{ij}$, $W_{ij}$ as the adjacency matrix of the online social network, the offline mobility network, and the movement probabilistic network. Then, we use the following weighted linear combination to infer the actual movement patterns among MSM.

2.1 $$W_{ij}=\alpha \frac{X_{ij}}{X_{i}}+(1-\alpha )\frac{Y_{ij}}{Y_{i}},$$

where $X_{i}=\sum_{j}X_{ij}$, $Y_{j}=\sum_{i}Y_{ij}$, and α is the relative weight of the online social network in deciding the actual movement. Here, $W_{ij}$ indicates the movement preference for MSM in city i [24]. Specifically, if one of MSM in city i moves, he goes to city j with a probability $W_{ij}$. $W_{ii}=0$ and $\sum_{j}W_{ij}=1$. According to equation (2.1), we assume such movement preference is determined by the weight of the interaction on the online social network and the offline mobility network. Note that, although the online social network and the offline mobility network are both symmetric and undirected networks, i.e. $X_{ij}=X_{ji}$ and $Y_{ij}=Y_{ji}$, the movement probabilistic network is an asymmetric and directed network, i.e. $W_{ij}\neq W_{ji}$. Owing to limited data, we cannot obtain a specific value of α that describes the accurate combination of the online social network and the offline mobility network. We will present and compare the geographical spread patterns under different combinations (i.e. different values of α) in this study.

## 3. The Meta-Spudtr model

**Figure 3.**
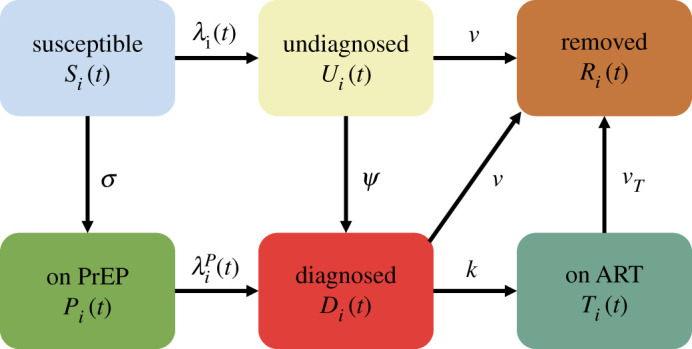
Illustration of the transitions between states within cities. Definitions for parameters in figure 3 are provided in the electronic supplementary material, equations (3.1) and (3.2). (Online version in colour.)

To investigate transmission patterns of a new HIV genotype within and between cities, we propose Meta-Spudtr, a metapopulation model where the whole MSM population in Guangdong is divided by a set of subpopulations connected by moving individuals (figure 3). To be specific, we follow the prefecture-level divisions, with each subpopulation corresponding to all MSM in a city. We describe the disease dynamics within each city based on a deterministic dynamic compartmental model in [25]. Individuals in each city are divided into six classes: susceptible, on PrEP, living with undiagnosed HIV, living with diagnosed HIV, on HIV ART, and removed. We illustrate the transitions between states (e.g. ‘Susceptible’, ‘On PrEP’, etc.) in figure 3.

Denote $S_{i}(t)$, $P_{i}(t)$, $U_{i}(t)$, $D_{i}(t)$, $T_{i}(t)$ and $R_{i}(t)$ as the number of susceptible, on pre-exposure PrEP, living with undiagnosed HIV, living with diagnosed HIV, on HIV ART, and removed individuals at time t for city i, respectively. $N_{i}=S_{i}(t)+P_{i}(t)+U_{i}(t)+D_{i}(t)+T_{i}(t)+R_{i}(t)$ is the total number of individuals in city i. Susceptible individuals will initiate PrEP at a rate σ and we assume that all individuals will not stop PrEP once starting. Susceptible and on PrEP individuals may acquire HIV through sexual activities with individuals living with undiagnosed HIV and diagnosed HIV. We hypothesize that the risks for transmitting HIV from people living with HIV but on regular ART treatment to susceptible individuals are minimal [26]. We also make an assumption that once diagnosed as HIV-positive, individuals will decrease the number of sexual contacts and use condoms in sexual activities. Denote the rates at which susceptible and on PrEP individuals acquire HIV are $\lambda_{i}(t)$ and $\lambda_{i}^{P}(t)$, respectively. For susceptible individuals,

3.1 $$\lambda_{i}(t)=C\beta_{S}\left[\frac{U_{i}(t)}{N_{i}}+(1-\theta )\;(1-\eta_{C})\frac{D_{i}(t)}{N_{i}}\right],$$

where C is the average number of contacts per person per time, $\beta_{S}$ is the transmission probability per anal sex act, θ is the relative decrease change in the number of contacts for individuals living with diagnosed HIV compared to those living with undiagnosed HIV, $\eta_{C}$ is the condom efficacy among MSM. Similarly, for individuals on PrEP,

3.2 $$\lambda_{i}^{P}(t)=C\beta_{P}\left[\frac{U_{i}(t)}{N_{i}}+(1-\theta )\;(1-\eta_{C})\frac{D_{i}(t)}{N_{i}}\right],$$

where $\beta_{P}=\eta_{P}\beta_{S}$ is the transmission probability per anal sex act with condom use. $\eta_{P}$ is the efficacy of PrEP. After being infected with HIV, on PrEP individuals will immediately become living with diagnosed HIV, while susceptible ones will first become undiagnosed and then transit to diagnosed at a rate ψ. The mortality rates for individuals living with undiagnosed HIV and diagnosed HIV are the same, denoted by v. Individuals living with diagnosed HIV will initiate ART at a rate k and we assume that no one will drop out once starting. The mortality rate for individuals on ART is denoted by $v_{T}$. In addition to transmission dynamics within each city, HIV can spread between cities through individual movements. Denote $\gamma =\sum_{i,j}Y_{ij}/\sum_{i}N_{i}$ as the average movement rate, i.e. each MSM decides whether or not to move with probability γ or remain in current city with probability 1−γ. If he moves, the choice of destination is decided by $W_{ij}$ obtained from equation (2.1). Then, we can obtain the differential equations to describe HIV transmission dynamics within and between cities as follows:

3.3 $$\begin{array}{rl} & \partial_{t}S_{i}(t)=-[\lambda_{i}(t)+\sigma ]S_{i}(t)+\gamma N_{i}\sum_{j\neq i}W_{ij}\left[\frac{S_{j}(t)}{N_{j}}-\frac{S_{i}(t)}{N_{i}}\right],\\ & \partial_{t}P_{i}(t)=\sigma S_{i}(t)-\lambda_{i}^{P}(t)P_{i}(t)+\gamma N_{i}\sum_{j\neq i}W_{ij}\left[\frac{P_{j}(t)}{N_{j}}-\frac{P_{i}(t)}{N_{i}}\right],\\ & \partial_{t}U_{i}(t)=\lambda_{i}(t)S_{i}(t)-(\psi +v)U_{i}(t)+\gamma N_{i}\sum_{j\neq i}W_{ij}\left[\frac{U_{j}(t)}{N_{j}}-\frac{U_{i}(t)}{N_{i}}\right],\\ & \partial_{t}D_{i}(t)=\psi U_{i}(t)+\lambda_{i}^{P}(t)P_{i}(t)-(k+v)D_{i}(t)+\gamma N_{i}\sum_{j\neq i}W_{ij}\left[\frac{D_{j}(t)}{N_{j}}-\frac{D_{i}(t)}{N_{i}}\right],\\ & \partial_{t}T_{i}(t)=kD_{i}(t)-v_{T}T_{i}(t)+\gamma N_{i}\sum_{j\neq i}W_{ij}\left[\frac{T_{j}(t)}{N_{j}}-\frac{T_{i}(t)}{N_{i}}\right]\\ \;\text{and}\; & \partial_{t}R_{i}(t)=v[U_{i}(t)+D_{i}(t)]+v_{T}T_{i}(t)+\gamma N_{i}\sum_{j\neq i}W_{ij}\left[\frac{R_{j}(t)}{N_{j}}-\frac{R_{i}(t)}{N_{i}}\right]. \end{array}\}$$

The model works in a monthly time step. Note that (a) there is no entry into or departure from the whole MSM population; (b) the model is restricted to the transmission of the new genotype of HIV, thus, infections with other genotypes are not considered; (c) we only consider HIV transmission among MSM (i.e. via anal sex) in this model for simplicity. The values of epidemiological parameters in equation (3.3) are estimated mainly based on previous sexual behavioural surveys and reports of MSM in Guangdong, as all cities in Guangdong province are selected for modelling. Corresponding references are provided in the electronic supplementary material.

## 4. Simulations and results

### (a) General transmission patterns

**Figure 4.**
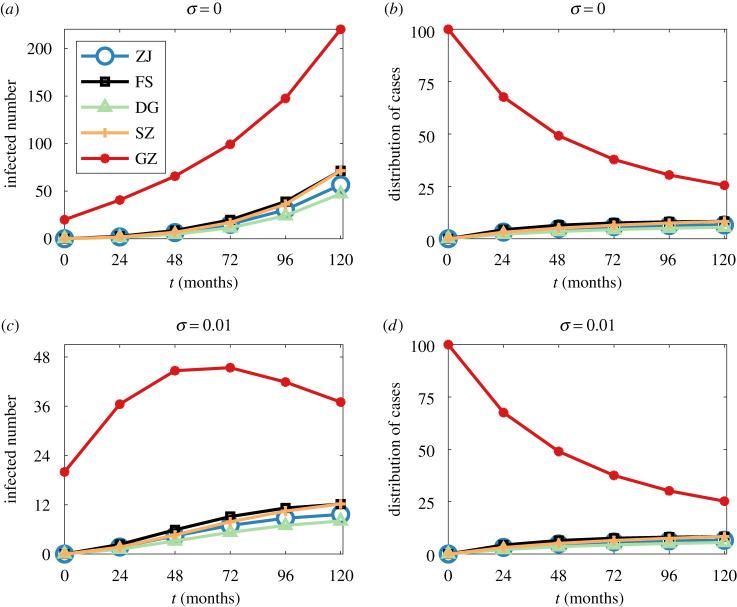
Total numbers of infected MSM (i.e. MSM living with HIV) in GZ, SZ, DG, FS and ZJ with (*a*) σ=0 and (*c*) σ=0.01. Distribution of infected MSM in GZ, SZ, DG, FS and ZJ with (*b*) σ=0 and (*d*) σ=0.01. α=0.5, Guangzhou is initially infected with 20 MSM at t=0. GZ, Guangzhou; SZ, Shenzhen; DG, Dongguan; FS, Foshan; ZJ, Zhanjiang. (Online version in colour.)

Figure 4 presents the total numbers of MSM living with HIV (i.e. undiagnosed, diagnosed but not on ART, and diagnosed and on ART) and the distribution of infected cases in the five most populous cities (i.e. Guangzhou, Shenzhen, Dongguan, Foshan and Zhanjiang) during the first 10 years with 20 MSM initially infected in Guangzhou. Although all five cities report infections within 2 years, most new infections still occur in the initial outbreak city, Guangzhou, within the first 10 years. The growth rate of infected cases for each city is partially relevant to the movement probability from the initial outbreak city to other cities: the movement probability from Guangzhou to Foshan (0.23) is greater than that to Dongguan (0.11), thus, the growth rate at Foshan is higher than that at Dongguan. Of note, the movement probabilities from Guangzhou to Dongguan and Shenzhen are almost the same, but we observe a slightly higher growth rate in Shenzhen than that in Dongguan. Besides, PrEP initiation has a significant effect in preventing infections: the number of infected cases in Guangzhou decreases gradually from the fifth year when σ is 0.01, while it keeps growing when σ is 0. Although the numbers of infected cases in other cities still grow when σ is 0.01, we can observe a much lower increase compared to the scenario when σ is 0 and the increase rate is getting slower. Details about the difference in the time series of infected cases are presented in the electronic supplementary material.

### (b) Impact of PrEP

To quantitatively explain the impact of PrEP and the dynamics underlying the geographical spread of HIV, we provide a simpler framework to capture the dynamics in equation (3.3). Essentially, if the PrEP initiation rate σ=0, the Meta-Spudtr model is approximately an SIR based metapopulation model. Previous studies have proposed a framework that can reduce the complex spatiotemporal patterns of such disease transmission by a simple propagation pattern. Specifically, the disease arrival time can be predicted by a probabilistically motivated effective distance [9,27]. Following this literature, we define the effective distance from city i to a connected city j as

4.1 $$d_{ij}=(1-\log W_{ij})\geq 1.$$

Note that, although the definition of $W_{ij}$ is a bit different from that in [9], they both quantify the fraction of individuals with destination city j emanating from city i. Equation (4.1) is consistent with the concept in [9] that a small $W_{ij}$ is equivalent to a large effective distance, and vice versa. Since $W_{ij}\neq W_{ji}$, then $d_{ij}\neq d_{ji}$. Then, we can obtain the length Λ(Γ) (based on the effective distance) of a directed path $\Gamma =\{i_{1},\ldots ,i_{L}\}$ as

4.2 $$\Lambda (\Gamma )=\sum_{m=1}^{m=L-1}d_{i_{m}i_{m}+1},$$

Then, we define the effective distance from city i to an arbitrary city j as the shortest length among all directed paths from city i to city j, i.e.

4.3 $$D_{ij}=\min_{\Gamma }\Lambda (\Gamma ).$$

Assuming the initial outbreak city of a specific genotype of HIV is city i, this genotype arrives in an arbitrary city j at time $T_{ij}^{a}$, the framework in [9] suggests a linear relationship between the disease arrival time $T_{ij}^{a}$ and the effective distance $D_{ij}$, i.e.

4.4 $$T_{ij}^{a}\propto D_{ij}.$$

**Figure 5.**
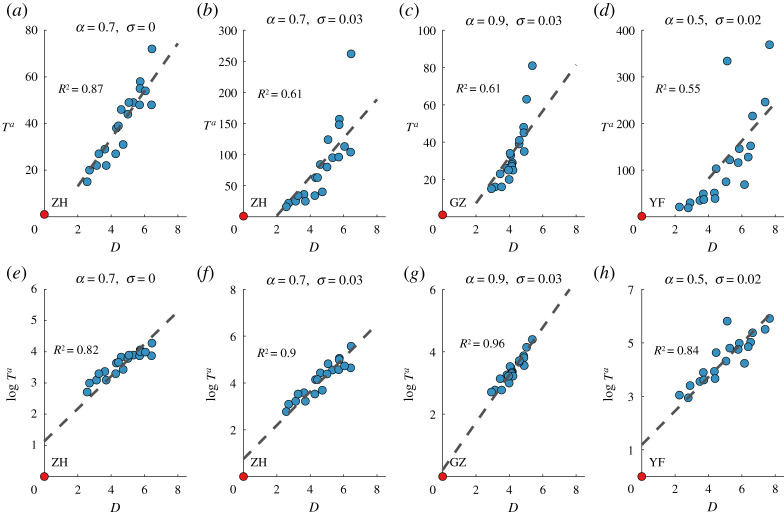
The relationship between the HIV arrival time $T^{a}$ and the effective distance D when initial outbreak city is Zhuhai, α=0.7, σ=0 in (*a*) and (*e*); initial outbreak city is Zhuhai, α=0.7, σ=0.03 in (*b*) and (*f*); initial outbreak city is Guangzhou, α=0.9, σ=0.03 in (*c*) and (*g*); initial outbreak city is Yunfu, α=0.5, σ=0.02 in (*d*) and (*h*). The number of initially infected MSM is 20. GZ,Guangzhou; ZH,Zhuhai and YF,Yunfu. (Online version in colour.)

The arrival time is defined as the date of the first infected individual (living with undiagnosed or diagnosed HIV). Figure 5*a* demonstrates the relationship between $T^{a}$ and D with the initial outbreak in Zhuhai, α=0.7, and σ=0. We observe a high value of $R^{2}$, indicating that the HIV transmission patterns can be well characterized by equation (4.4) with no one taking PrEP. However, as σ grows, $T^{a}$ correlates weakly with D. Figure 5*b* demonstrates the relationship between $T^{a}$ and D when σ=0.03, other parameters are the same as that in figure 5*a*. We observe that $R^{2}$ decreases greatly, dropping to 0.61 in figure 5*b* from 0.87 in figure 5*a*. This indicates that, since the framework in [9] did not consider the impact of prevention tools, equation (4.4) cannot perfectly characterize the underlying dynamics of HIV transmission as σ grows.

Here, we propose a new framework that can better capture the geographical spread patterns of HIV among MSM considering the impact of PrEP. We modified the framework in [9] to characterize the relationship between the disease arrival time $T_{ij}^{a}$ and the effective distance $D_{ij}$ as

4.5 $$\log T_{ij}^{a}\propto D_{ij}.$$

Figure 5 demonstrates the comparison of equations (4.4) and (4.5) in describing the relationship between the arrival time $T^{a}$ and the effective distance D with different initial outbreak cities, different combinations of online social network and offline mobility network, and different PrEP initiation rates. We can observe in all subfigures that, due to the impact of PrEP (σ>0), effective distance D has a stronger correlation with the natural logarithm of the arrival time $\log T^{a}$ than the arrival time $T^{a}$ itself. More similar results for a wide range of σ are presented in the electronic supplementary material. These indicate that (a) given an initial outbreak city, the order in which each city is infected by HIV transmission depends only on the underlying movement probabilistic network regardless of the PrEP initiation rate σ; (b) PrEP initiation exponentially delays the occurrence of a specific genotype of HIV for all cities.

### (c) ‘Earliest’ exposure risk

According to the linear relationship between $\log T^{a}$ and D, virus occurrence delays are much shorter in cities with shorter effective distances from the initial outbreak city. Thus, cities with shorter distances from other cities remain at high risk of immediate exposure to the virus, even with the impact of PrEP. An earlier occurrence of the virus indicates a shorter time for preparedness against the virus. Here, we define the ‘earliest’ exposure risk for each city by the probability of being the first infected city except the initial outbreak city. Given a movement probabilistic network (fixed α), the ‘earliest’ infection risk for city i ${\text{EIR}}_{i}$ is denoted as

**Figure 6.**
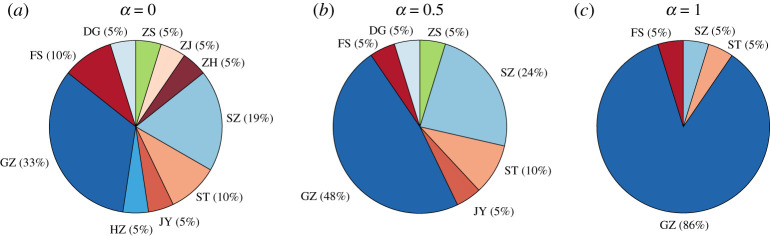
The ‘earliest’ exposure risk for each city with respect to (*a*) α=0, (*b*) α=0.5, and (c) α=1. Cities with $EIR_{i}=0$ are not presented in the figure. GZ, Guangzhou; ZH, Zhuhai; HZ, Huizhou; JY, Jieyang; ST, Shantou; SZ, Shenzhen; ZH, Zhuhai; ZJ, Zhanjiang; ZS, Zhongshan; DG, Dongguan; FS, Foshan. (Online version in colour.)

**Figure 7.**
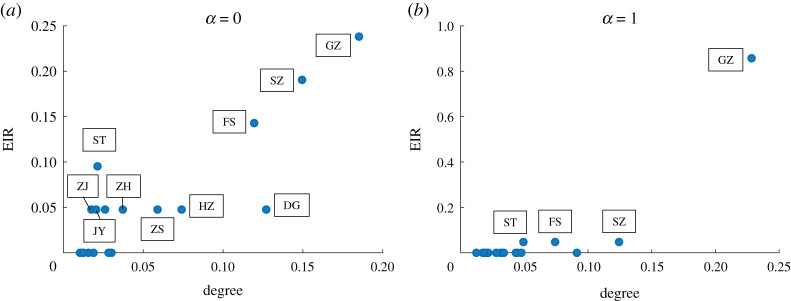
Relationship between EIR and the weighted degree for each city with (*a*) α=0 and (*b*) α=1. The weighted degree for each city is rescaled by dividing through the sum of the weighted degree of all cities. GZ, Guangzhou; ZH, Zhuhai; HZ, Huizhou; JY, Jieyang; ST, Shantou; SZ, Shenzhen; ZH, Zhuhai; ZJ, Zhanjiang; ZS, Zhongshan; DG, Dongguan; FS, Foshan. (Online version in colour.)

4.6 $${\text{EIR}}_{i}=\frac{\sum_{j}I(D_{ji}={\text{min}}_{j\neq k}D_{jk})}{n},$$

where n is the number of cities, I(⋅) is the indicator function. $\sum_{i}{\text{EIR}}_{i}=1$. Figure 6 illustrates the ‘earliest’ exposure risk for each city with respect to different values of α. We can observe that Guangzhou, Shenzhen, Foshan and Shantou will always face the risk of ‘earliest’ exposure regardless of the value of α. The differences in the ‘earliest’ exposure risks for cities can also be well explained by their positions on the movement network. Here, we plot the relationship between EIR and the weighted degree for each city with α=0 and α=1, respectively, in figure 7. We can observe in both subfigures that hub cities usually face higher ‘earliest’ exposure risk. As α grows, fewer cities have large degrees while Guangzhou becomes a larger hub, thus, the ‘earliest’ exposure risk has been transferred to Guangzhou from other cities. Compared to figure 6*a*, we can observe that fewer cities face the ‘earliest’ exposure risk while Guangzhou faces a much higher ‘earliest’ exposure risk in figure 6*c*.

### (d) Most possible transmission path

**Figure 8.**
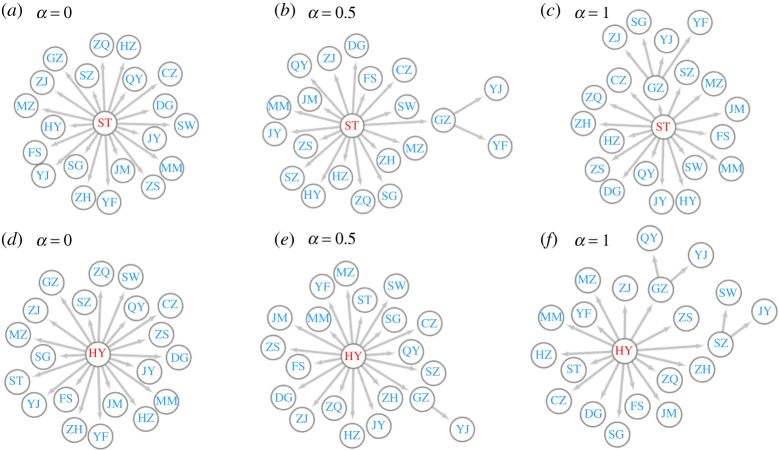
The shortest path tree for (*a*) α=0, initial outbreak in ST, (*b*) α=0.5, initial outbreak in ST, (*c*) α=1, initial outbreak in ST, (*d*) α=0, initial outbreak in HY, (*e*) α=0.5, initial outbreak in HY, and (*f*) α=1, initial outbreak in HY. ST,Shantou; HY,Heyuan. (Online version in colour.)

**Figure 9.**
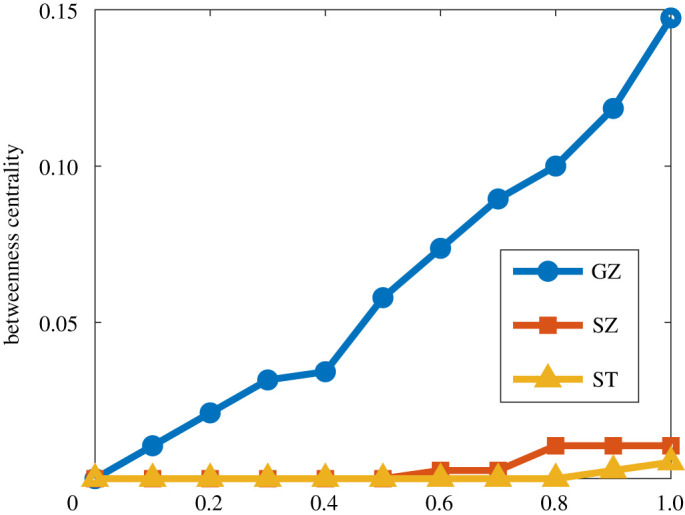
Betweenness centrality for GZ (Guangzhou), SZ (Shenzhen) and ST (Shantou) on the effective distance-based movement network. Other cities with betweenness centralities of zero are not presented here. (Online version in colour.)

By converting weights of edges on the movement probabilistic network to the effective distance based on equation (4.1), we can obtain an effective distance-based movement network among MSM. The shortest path tree generating from the effective distance-based movement network indicates the most possible transmission path from the initial outbreak city to a random city. Figure 8 illustrates the shortest path tree with different values of α and different initial outbreak cities. Here, we only present the results for α∈{0,0.5,1} and initial outbreak in {ST (Shantou), HY (Heyuan)}. Results for other scenarios are consistent with the results in figure 8. We can observe that most cities acquire the virus directly from the initial outbreak city, while some cities acquire the virus from non-initial outbreak cities like Guangzhou, Shenzhen and Shantou. Figure 9 illustrates the betweenness centralities [28] for these three cities on the effective distance-based movement network with different values of α. As α increases, all betweenness centralities for Guangzhou, Shenzhen and Shantou increase. Besides, Guangzhou always has a higher betweenness centrality than Shenzhen and Shantou. These results quantitatively demonstrate the importance of each city in facilitating HIV transmission between cities. Containment of the outbreak in cities with high betweenness centralities slows the contagion of the virus, because several cities can only reach each other via longer paths. It is not surprising that Guangzhou, the capital of Guangdong province and Shenzhen, the first special economic zone in China, play a huge role in bridging cities and facilitating transmission. However, Shantou, as a medium sized city in Guangdong province, should also not be neglected for control and prevention measures.

## 5. Conclusion

In this study, we have developed the Meta-Spudtr, a metapopulation model considering the impact of PrEP, to investigate the transmission patterns of a specific genotype of HIV among MSM in Guangdong, China. Our Meta-Spudtr model works based on an inter-city movement probabilistic network among MSM, which integrates the offline mobility network and the online social network among MSM. Based on previous studies [9], we propose a new framework that can quantitatively capture the geographical spread patterns of HIV under the circumstances when PrEP services are provided to MSM.

We find that (a) although the virus arrives in some cities 2 years after the initial outbreak, the majority of new infections remain in the initial outbreak city in the first 10 years; (b) PrEP initiation will not change the order in which cities will be infected; (c) but PrEP initiation exponentially delays the occurrence of a new HIV genotype in all rest cities transmitted from the outbreak city; (d) due to the hub-effect, Guangzhou, Shenzhen, Foshan and Shantou will always face the risk of ‘earliest’ exposure in all combinations of the offline mobility network and the online social network; (e) most cities acquire the virus directly from the initial outbreak city, while some cities acquire the virus from cities that are not initial outbreak locations but have relatively high betweenness centralities on the effective distance-based movement network, such as Guangzhou, Shenzhen and Shantou. Our study extends previous studies [9] to predict the arrival times of a new HIV genotype considering the impact of PrEP. It also assesses the exposure risk and measures the importance in facilitating transmission for each city in Guangdong. This framework can be easily extended to investigate the large-scale transmission patterns of other infectious diseases with the impact of intervention tools by quantifying the underlying movement probabilistic network and the related epidemiological parameters. Our model has limitations. First, we do not consider the heterogeneity in PrEP initiation rates, HIV testing rates, ART initiation rates among cities, and the sexual behaviour preferences, contact patterns among MSM. Incorporating such heterogeneity will be interesting future work. Second, due to limited access to administrative data, we combine online social networking apps and public website documenting human mobility for estimation, and the value of α cannot be accurately determined. Further studies should be conducted for calibrations of the current model in real-world applications.

(All identifiable personal information was removed for privacy protection.)

## Data accessibility

Some parts of our data are available after signing a confidential agreement. For more information about the data access, contact our two corresponding authors.
